# A compendium of causative agents of occupational asthma

**DOI:** 10.1186/1745-6673-8-15

**Published:** 2013-05-24

**Authors:** Xaver Baur

**Affiliations:** 1 Institute for Occupational Medicine, Charité University Medicine Berlin and EOM Society, Berlin, Germany

## Abstract

**Objective:**

The objective is to provide an evidence-based compendium of allergenic and irritant agents that are known to cause occupational asthma in order to improve diagnostics and disease management.

**Methods:**

Two previously published reviews from our group utilized database searches to identify studies which were then rated according to the Scottish Intercollegiate Guideline Network (SIGN) grading system. The evidence level for each causative agent or worksite was graded using the Royal College of General Practitioners (RCGP) three-star system.

**Results:**

Approximately 3,000 relevant papers were identified, which covered 372 different causes of allergic and 184 different causes of irritant occupational asthma. The highest level achieved using the SIGN grading system was 2++, indicating a high quality study with a very low risk of confounding or bias and a high probability of a causal relationship. Using the modified RCGP three-star grading system, the strongest evidence of association with an individual agent or worksite ('***') was found for exposure to laboratory animals. Associations with moderate evidence level (‘**’) were obtained for a) the allergenic agents or worksites: alpha-amylase from *Aspergillus oryzae*, various enzymes from *Bacillus subtilis*, papain, bakeries, western red cedar, latex, psyllium, storage mites, rat, carmine, egg proteins, Atlantic salmon, fishmeal, Norway lobster, prawn, snow crab, seafood, trout and turbot, reactive dyes, b) the irritant agents or worksites: benzene-1,2,4-tricarboxylic acid, 1,2- anhydride [trimellitic anhydride], chlorine, cobalt, cement, environmental tobacco smoke, grain, welding fumes, construction work, swine confinement, World Trade Center disaster 2001, and c) agents or worksites causing allergic as well as irritant occupational asthma, included farming, poultry confinement, various isocyanates and platinum salts. A low evidence level (RCGP) was obtained for 84 agents or worksites (42 from each group), providing a total of 141 conditions with a low, moderate or strong evidence level.

**Conclusion:**

This work comprises the largest compendium and evaluation of agents and worksites causing allergic or irritant occupational asthma from the literature assessed in an evidence-based manner.

## Introduction

**Figure 1:**
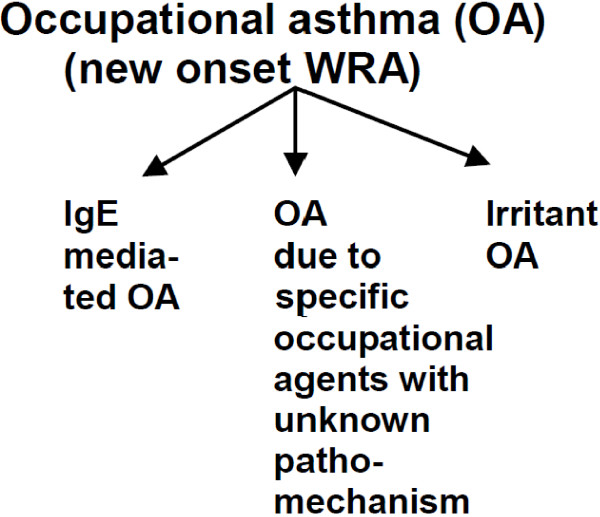
Subgroups of occupational asthma.

*Occupational asthma* is a disease characterized by variable airflow limitation and/or hyper-responsiveness associated with inflammation which is attributable to causes and conditions in a particular occupational environment and not to stimuli encountered outside the workplace [1]. Occupational asthma involves IgE-mediated asthma after a latency period, irritant asthma with or without a latency period, including reactive airways dysfunction syndrome (RADS), resulting from high exposure, and asthma due to specific occupational agents with unknown pathomechanisms that may also show a latency period [2,3] (Figure 1). Occupational asthma needs to be differentiated from *work-aggravated asthma,* which is characterized by a worsening of pre-existing asthma or increase in airway resistance, asthma medications or frequency and/or severity of asthma attacks arising from causes and conditions attributable to a particular occupational environment and not to stimuli encountered outside the workplace. These workers have a concurrent history of asthma that was not induced by exposure in the workplace. Aggravation is typically due to an occupational irritant (e.g. non-sensitizing fumes) [4]. There are also workers with pre-existing asthma who, after a latent interval, have a worsening of their pre-existing non-occupational asthma after regular daily exposure to agents which cause IgE-mediated allergies. No detailed studies or valid statistics relevant to work-aggravated asthma are available. The current study focuses on the identification of causes of occupational asthma and is based on two recently published reviews, evaluating occupational allergens [5] and irritants [6] in an evidence-based manner.

## Methods

Appropriate terms were used to search Medline via PubMed (http://www.ncbi.nlm.nih.gov/mesh). The combination of medical subject headings (MeSH) used were as follows

### Occupational asthma due to allergens

("1"[PDAT] : "2011/08/01"[PDAT]) AND ((((((((((("Signs and Symptoms, Respiratory"[MeSH] OR "Occupational Diseases"[MeSH]) OR "Allergy and Immunology"[Majr]) OR "Respiratory Function Tests"[MeSH]) OR "Bronchial Hyperreactivity"[MeSH]) OR "Airway Obstruction"[MeSH]) OR "Respiratory Hypersensitivity"[MeSH]) OR "Lung Diseases, Obstructive"[MeSH]) OR "Asthma"[MeSH]) OR "Asthma"[Mesh]) OR "Occupational Exposure"[MeSH]) AND "ALLERGEN"[MeSH]) AND ("humans"[MeSH] AND (English[lang] OR French[lang] OR German[lang]) AND "adult"[MeSH])

### Occupational asthma due to irritants

This procedure differed from the aforementioned with "Respiratory Hypersensitivity"[MeSH]), "ALLERGEN"[MeSH]) replaced by "Asthma/chemically induced"[MeSH], "Lung Diseases, Obstructive/*chemically induced"[MeSH], "Occupational Diseases/chemically induced"[MeSH].

The searches were completed and included publications up until August 2011 (allergens) and to the end of June 2012 (irritants).

References collated in seminal systematic reviews or overviews of causes of work-related asthma were also included and the results from both approaches were combined.

The occupational diseases statistics based either on statutory surveillance or registration systems: SWORD 1994–1997 [7-9], SHIELD 1993 [10], SORDSA 2001 [11], SENSOR [12] and Dokumentation der Berufskrankheiten 2007 (BK-DOK) [13], were also included.

A list of agents denoted as “may cause sensitization by inhalation“ by the phrase H334 (until 2011 R42), and as “may cause respiratory irritation” by the phrase H 335 (until 2011 R37), respectively, according to regulations of the European Parliament [14] was also considered.

All studies were assessed for potential biases (selection, confounding, information bias), in particular the sources of bias and bias minimisation strategies in either the design or analysis phase, specific to each study design.

The methodological quality of each selected publication was assessed by an experienced reviewer and discussed with the senior physician of the occupational unit who independently assessed the publications, before rating each according to the modified Scottish Intercollegiate Guidelines Network (SIGN) classification [15]. The slightly extended RCGP (Royal College of General Practitioners 1995) three-star system was used to grade the evidence for each agent on the basis of all available publications. The three-star system considers quality and quantity of all studies as well as consistency of reported findings. Our extensions comprised: [ ] indicated down-grading due to lower quality of clinical investigations, i.e., due to missing objective parameters such as lung-function data; further, (*) indicated up-grading from '-' due to at least 5 reported asthma cases without contradictory findings (see Table 1). For further methodological details of the selection criteria, data extraction and synthesis, quality assessment of individual studies and the full list of ratings for each publication, see [5,6].

**Table 1:** The modified Royal College of General Practitioners (RCGP) three-star system of the British Occupational Health Research Foundation


***	Strong evidence – provided by generally consistent findings in multiple, high quality, scientific studies.
**	Moderate evidence – provided by generally consistent findings in fewer, smaller or lower quality, scientific studies.
*[*]	Moderate evidence – provided by generally consistent findings in fewer, smaller or lower quality, scientific studies, based on questionnaire-conducted studies or other weak evidence (i.e. clinical weakness due to absence of LFT, sPFT, SIC)
*	Limited or contradictory evidence – provided by one scientific study (analytic) or inconsistent findings in multiple scientific studies.
[*]	Limited or contradictory evidence – provided by one scientific study (analytic) or inconsistent findings in multiple scientific studies, based on questionnaires or other weak evidence (i.e. clinical weakness due to absence of LFT, sPFT, SIC)
(*)	Very limited or contradictory evidence – provided by at least three case reports, one case series, one non-analytic study or one occupational disease statistical study with at least five asthma cases
-	No scientific evidence – based on clinical studies, theoretical considerations and/or clinical consensus

(BOHRF, [16]) Applied modifications are underlined.

## Results

### **Overview** (Table 2)

**Table 2:** Review of the literature on allergens or irritants causing occupational asthma

**Findings**	**Allergens**	**Irritants**
Potentially relevant publications and considered	2,315	863
Publications which were found to be useful and were included	865	474
Different potential causative agent or workplaces identified	372	184
Evidence-based causative agents or workplaces*	78	71

*Overlap of 12 agents resulting of 137 different causative allergenic and/or irritant causative agents or workplaces.

The database search resulted in 2,315 potentially relevant publications referring to allergen-induced asthma and 863 referring to irritant-induced asthma. After exclusion of non-occupational cases, a total of 865 allergen-induced and 474 irritant-induced asthma studies remained. These publications refer to 372 individual allergenic and 184 irritant agents or worksites that were identified to cause occupational asthma, of which 36 were listed as both allergens and irritants, leaving a final total of 520 different causes of occupational asthma. For 78 allergens and 71 irritants, (of which 12 were in both groups), an evidence level of at least ‘*’ in the RCGP grading system was found.

### **Strength of evidence per agent or worksite** (Table 3)

**Table 3:** Strength of evidence for occupational asthma-caused by allergens, irritants or worksites according to the modified RCGP three-star system

**Evidence level (modified RCGP three star grading)**	**Allergens /irritants: number of agents or worksites**	**Agent or worksite allergen**	**Agent or worksite irritant**
			**[Synonym] (CAS)**
***	1/0	Co-exposure to various lab animals	-
**	18/12	Alpha-amylase from *Aspergillus oryzae*; various enzymes from *Bacillus subtilis;* papain; bakery (flour; amylase; storage mites); western red cedar; latex; *Psyllium*; farming (animals, cereal, hay, straw and storage mites); storage mites; rats; carmine; egg proteins; Atlantic salmon; fishmeal; Norway lobster; prawns; snow crabs; seafood; trout and turbot; reactive dyes; toluene diisocyanates (TDI); platinum salts;	Benzene-1,2,4-tricarboxylic acid; 1,2- anhydride [trimellitic anhydride] (552-30-7); chlorine (7782-50-5); cobalt (7440-48-4); various isocyanates, isocyanurate (HDI, MDI, TDI), phenylmethane diisocyanate [MDI] (5873-54-1), toluene diisocyanate, TDI 2,4 (584-84-9),TDI 2,6: (91-08-7); platinum salts (7440-06-4); cement [78]; environmental tobacco smoke; grain [78]; welding fumes; construction work (dust, agent unspecified); farming, animals (pig, beef/veal, dairy, poultry); swine confinement; World Trade Center disaster 2001
*[*]	17/39	Detergent enzymes*,* soybean (husks, flour); paprika; tea dust; tobacco; *Aspergillus niger*; cows; predatory mites; spider mites; opiates; methylene diphenyl diisocyanate (MDI), phthalic anhydrides; various isocyanates	Ceramic production [78]; phthalic anhydride (85-44-9); glutaraldehyde [glutaral] (11-30-8); sulfur dioxide (7446-09-5); cotton (dust; raw) CNT 750; potroom aluminum smelting; farming (various); foundry; smoke (fires, pyrolysis products); pesticides (unspecified); cleaning agents (unspecified); health care workers;
*	18/39	Eastern white cedar; various flowers; guar gum; poppy; rose (*Rosa rugosa*); senna; ispaghula husks; sunflower pollen; trypsin; various woods (abies, chestnut, douglas, framire, mansonia, oak, obeche, walnut, white poplar); weeping fig; nonbiting midges; hexahydrophthalic anhydride; hexamethylene diisocyanate (HDI); methyl tetrahydrophthalic anhydride (MTHPA); persulfate salts; polyfunctional aziridine	Acetic acid (64-19-7); sulfuric acid (7664-93-9); metacrylates; loctide (53858-53-0); aluminum salts [aluminum fluoride] (7724-18-1); aluminum sulfate: (10043-01-3); ammonia (7664-41-7); various anhydrides; tetrachlorophthalic anhydride (117-08-8); azobisformamide (123-77-3); cadmium (fumes) (7440-43-9); carbon black dust (1333-86-4); ethylenediamine (107-15-3); formaldehyde (gas, dust) (50-00-0); hexamethylene-tetramine (100-97-0); methyl isocyanate [MIC] (624-83-9); naphthylene diisocyanate (3173-72-6); polymethylene polyphenyl isocyanate (9016-87-9); N-methylmorpholine (09-02-4); ozone (gassings) (10028-15-6); paraquat (4685-14-7); diammonium peroxodisulfate (7727-54-0); phenylglycine acid chloride (39478-47-2); piperazine dihydrochloride (142-64-3); polyvinyl chloride (fume) (9002-86-2); rosin core solder; thermal decomposition (8050-09-7); vanadium (7440-62-2) + divanadium pentoxide (1314-62-1); cleaning agents (not specified); green coffee [78]; diesel exhaust; endotoxin; oil (spill); paint (fumes); pesticides (unspecified); reactive dyes; refractory ceramic fibers [RCF]; smoke (fires, pyrolysis products; oil fire and dust storm); soldering flux; solvents (unspecified); healthcare workers; poultry confinement; slaughter house; metal industry workers
[*]	24/3	Alternaria; bromelain from *Ananas comosus;* cellulase from *Trichoderma viride*; lactase from aspergillus; various enzymes; chrysanthemums; castor beans; madagascar jasmine; pine; flowers; budgerigar; flour moth; house dust mites; mouse; poultry; red soft corals; screw-worm fly; shrimp; various birds; cephalosporine; penicillins; phenylglycine; acid chloride; trimellitic anhydride	Nitrogen chloride (10025-85-1); polyamines; aliphatic; potassium persulfate (7727-21-1) and ammonium peroxydisulfate (7727-54-0); grain rice [78]
(*)	19/29	Aspergillus enzymes; cellulase from *Trichoderma reesei;* pancreatin; proteolytic enzymes*;* asparagus; *Boletus edulis*; carnation; garlic dust; rye flour; gum arabic; iroko; various woods; African maple; black bat; mealworm; poultry mites; tetrachlorophtalic anhydride; chloramine T; chromium and nickel	Acids not specified; hydrochloric acids (7647-01-0); alkyl cyanoacrylates; 3-amino-5-mercapto-1;2,4-triazole l(16691-43-3); aziridine, polyfunctional [78] (64265-57-2); chloramine T (powder dust) (7080-50-4); chromate (not specified); 3-(diamino-amino)propylamine (109-55-7); dichlorodiethyl sulfide (505-60-2); 2-diethylaminoethanol (100-37-8); diinitrogen tetraoxide (10544-72-6); hexamethylene diisocyanate [HDI], plus isodurane diisocyanate (822-06-0); HDI biuret plus (4035-89-6); anhydrous nickel sulfate (7786-81-4); hexahydrate (10101-97-0); paraphenylenediamine (106-50-3); persulfate (not specified); polypropylene, heated to 250°C (9003-07-0); potassium dichromate (7778-50-9); potassium aluminum tetrafluoride (14484-69-6); sodium iso-nonanoyl oxybenzene sulfonate [SINOS] (123354-92-7); sodium metabisulfite (7681-57-4); styrene monomer (100-42-5); chlorofluorocarbons (degradation products); hairdressing chemicals; lubricants (unspecified); paper dust A111; aliphatic polyamines; polyester; powder paints; smoke (biomass, indoor)
-	275/94	Unlisted as no corroborating scientific evidence presented	Unlisted as no corroborating scientific evidence presented

*CAS,* Chemical abstracts service.

*RCGP,* Royal College of General Practitioners.

#### Allergens

Strong evidence, i.e. three stars ‘***’ was achieved from exposure to laboratory animals. For 18 agents or worksites, the strength of evidence corresponded to two stars ’**’. Moderate evidence, provided by generally consistent findings in fewer, smaller or lower quality scientific studies with clinical weakness ‘*[*]’ was found for 17 agents. Limited or contradictory evidence ‘*’, was identified for 18 agents. For 24 agents, down-grading to '[*]' was necessary because of absent objective data.

The majority of agents were investigated in non-analytical studies. Thus, the strength of evidence ranged from very limited or contradictory evidence ‘(*)’ (19 agents or worksites) to no scientific evidence ‘-‘ (275 agents or worksites).

#### Irritants

For 17 (mixed) agents or worksites, two stars ‘**’ were achieved and none reached a higher level. Low to moderate scientific evidence, i.e. ‘*[*]’ , was found for 12 agents. Limited or contradictory evidence.’*’ was identified for 39 agents or worksites and a further three agents were down-graded because of inadequate methodological aspects. "Very limited or contradictory evidence” was obtained 29 times and no scientific evidence 94 times.

Interestingly, our literature search identified a group of 15 irritant agents or worksites that were found to cause occupational asthma and/or COPD, namely: ammonia, cement dust, chlorine, cleaning agents, mustard gas, diesel exhaust, environmental tobacco smoke, isocyanates, smoke from fires, sulfur dioxide, construction work, swine confinement, farming, foundry, metallurgic industry.

#### Agents behaving as both allergen and irritant

36 agents or worksites were found to cause allergic as well as irritant occupational asthma; for twelve of them evidence – mainly at a low level – was available (Table 4). With the exception of anhydrides and isocyanates there was no close relationship with the respective H334 and H335 phrases, respectively.

**Table 4:** **36 agents identified as occupational asthma-inducing allergens as well as irritants (see column 1 of Table**3**for full range of evidence levels)**

**Agent**	**Evidence level as allergen**	**Evidence level as irritant**	**H334**	**H335**
Aluminium/potroom	-	*		
*2-Ethanolamine*	*-*	*-*		x
*Aminoethylethanolamine*	*-*	*-*		x
*Anhydrides, various*	*-*	*	x	
*Anhydrides, hexahydrophthalic*	*	*-*	x	
*Anhydrides, maleic*	*-*	*-*	x	
*Anhydrides, methyltetrahydrophththalic*	*-*	*-*	x	
Anhydrides, phthalic anhydride	*[*]	*[*]	x	x
Anhydrides, tetrachlorophthalic anhydride	(*)	*	x	
Azobisformamide	-	*	x	
Captafol	-	-		
Chloramine T	(*)	(*)	x	
Chlorohexidine	-	-	x	x
Cobalt	-	**		
Diethanolamine	-	-		
Formaldehyde	-	*		x
Glutaraldehyde	-	*[*]	x	
Hexachlorphene	-	-		
Isocyanate, diphenylmethane diisocyanate (MDI)	*[*]	**	x	x
Isocyanate, hexamethylene diisocyanate (HDI)	*	(*)	x	x
Isocyanate, 1,5-naphthylene diisocyanate (NDI)	-	(*)	x	x
Isocyanate, toluene diisocyanate (TDI)	**	**	x	x
Isocyanate, triglycidil isocyanurate	-	-		
Paraphenylenediamine	-	(*)		
Persulfate	*	(*)	x	x
Piperazine dihydrochloride	-	*	x	
Platinum salts	**	**		
Tetrachloroisophthalonitrile	-	-		x
Tributyl tin oxide	-	-		
Vanadium	-	*		
Zinc	-	-		
Green coffee (dust)	*[*]	*		
Reactive dye	**	*		
Welding fumes	-	**		
Farming	**	*[*]		
Poultry confinement	[*]	*(*)		

## Discussion

As reported by various authors ([17-19]), about 15% of adult asthma cases are caused by occupational agents, whether IgE-mediated or irritant occupational asthma or occupational asthma due to unknown pathomechanism. Irritant asthma may arise from high dose, moderate or even low dose exposure with the clinical pictures of RADS and not-so-sudden-onset irritant asthma. The latter, as well as occupational asthma of unknown pathomechanism, mostly show a latency period from the beginning of causative exposure until the appearance of symptoms, which makes these indistinguishable from allergic asthma [20].

Previous reports and reviews have accumulated narrative and experimental evidence for about 300 causative agents, which are often divided into high or low molecular weight groups ([21-28]). High molecular weight agents typically induce asthma through an IgE-mediated mechanism while the pathomechanism of low molecular weight agents is mostly airway irritation or unknown.

Quirce and Sastre recently summarised the newer causative agents published between 2009 and 2011 [22]. A continuously updated classification of allergenic or irritant occupational asthma agents has been provided by the American Conference of Governmental Industrial Hygienists (ACGIH) [29], the German MAK commission [30], the Health and Safety Executive [31] and the European Community [14]. At present, the new international labelling of working materials hazardous to health (GHS; EU: CLP), which includes airway allergenic and iritative agents, has been put in force and has replaced the hitherto R and S notes with hazard (H) and precautionary statements (P) as well as by new hazard pictograms (European Parliament [14], United Nations [32]).

These reviews and classifications all lack an evidence-based evaluation of identified agents in the clinical literature. An evidence-based evaluation of the literature is of particular importance for case management and diagnostics in clinical practice. Thus, the objective of this work was to provide a scientific compendium of practical relevance, exhaustively listing the agents and worksites known to cause occupational asthma.

From more than 3,000 publications, 1,339 were retrieved from our Medline/PubMed and additional database searches, which refer to 372 individual agents or worksites that have been identified to cause allergic occupational asthma and 184 individual agents or worksites known to cause irritant occupational asthma. Of these 520 individual agents or worksites, 36 occurred in more than one category.

Evidence for causing occupational asthma was found for 78 allergens and for 71 irritants, respectively, with 8 eliciting an allergic as well as an irritative pathomechanism, establishing 141 in total.

The evidence levels for causing occupational asthma of many of the listed agents or worksites are moderate to low since only about a quarter of the identified studies were analytical, primarily because randomised controlled trials are not considered ethical when studying the exposure effects of harmful agents. Therefore, high quality studies are largely absent and the few available studies are frequently small in number of workers studied.

The vast majority are surveys, case series or case reports with evidence levels rated very low. The common diagnostic procedure for occupational asthma in clinical settings is a stepwise approach, including questionnaires about asthma-specific symptoms as well as respiratory and allergy assessments. Self-reported work-related symptoms are relatively sensitive indicators in the diagnosis of occupational asthma but the specificity is low. In approximately one third of the included studies, self-reported asthma symptoms or physician-reported asthma were used as the only diagnostic approach. Lung function, allergy testing and the diagnostic gold standards, serial spirometric or peak flow measurements (sPFT) or specific inhalation challenges, were each applied in only about one third to half of studies.

The level of evidence for single agents also depends on the absolute number of publications. Agents for which the research activity is higher may obtain higher levels in our rating system and, conversely, an absence or a low evidence grade of an occupational agent (e.g. in studies without SIC or lung function testing) does not necessarily exclude its potential for causing occupational asthma.

It is evident that more work is needed to consolidate the gaps, such as the study of potential asthma-inducing agents which have not or only rarely been investigated so far. This current overview provides a basis for such supplementary work and for surveillance programs of endangered workers. The latter may facilitate early diagnosis and the application of appropriate secondary preventive measures, leading to a significant reduction of asthma from identified causative exposure in the workplace.

## Competing interests

The author declares that he has no competing interests.
